# Peer Review of “Seroprevalence of SARS-CoV-2 in Niger State: Pilot Cross-Sectional Study”

**DOI:** 10.2196/50501

**Published:** 2023-10-17

**Authors:** ­ Anonymous

**Keywords:** COVID-19, pandemic, SARS-CoV-2, seroprevalence, serology, epidemiology, Niger State, Nigeria, COVID-19 testing, social distancing

*This is a peer-review report submitted for the paper “Seroprevalence of SARS-CoV-2 in Niger State: Pilot Cross-Sectional Study.”*

## Review Round 1

### General Comments

This paper, “Seroprevalence of COVID-19 in Niger State: A Pilot Cross-Sectional Study” by Majiya et al [1], is valuable and worthy of publication. The paper describes the seroprevalence of COVID-19 in Niger State. The COVID-19 asymptomatic rate in the state was 46.81%. The study also observed that the chances of infection are almost the same for both urban and rural dwellers. Of great interest is the finding that health care workers and those who had contact with persons who traveled out of Nigeria in the last 6 months are twice as likely to be at risk of being infected with the virus. The paper is relevant and contributes to the knowledge of the epidemiology of the virus. However, one primary concern is that the information about the virus from which inferences were made in this paper seems outdated. There is a need for an update. Also, the work appears to be underpowered in terms of sample size.

### Specific Comments

1. The abstract is unusually extended; consider summarizing it, especially the results aspect.
2. There is a need for editing and restructuring some sentences.
3. Some long paragraphs have the same references; consider using other references as well.
4. Give a reference or definition for your sampling technique and probably describe how you achieved your sample size.
5. Avoid repeating the methodology in the Discussion session.
6. Add references to back up your inferences.
7. The authors should make inferences in light of observation and the literature; asymptomatic cases seem to foster community transmission. More so, isolation, quarantine, and lockdown, if need be, are some public health measures to halt transmission. I would instead advise that the authors make recommendations based on the data generated from the study.

## Review Round 2

### General Comments

This paper, “Seroprevalence of COVID-19 in Niger State: A Pilot Cross-Sectional Study,” is a credible addition to the body of knowledge about COVID-19 in Niger State and Nigeria as a whole. The Abstract has been refined, and the Discussion better articulated. The authors might want to consider reducing the Introduction to about one and a half pages, making it more concise.

### Specific Comments

1. The authors should read through the paper to adjust for typographical errors.
